# Synchronous intracellular delivery of EGFR-targeted antibody–drug conjugates by p38-mediated non-canonical endocytosis

**DOI:** 10.1038/s41598-022-15838-8

**Published:** 2022-07-07

**Authors:** Jun-ichiro Takahashi, Shiori Nakamura, Iimi Onuma, Yue Zhou, Satoru Yokoyama, Hiroaki Sakurai

**Affiliations:** Department of Cancer Cell Biology, Faculty of Pharmaceutical Sciences, University of Toyama, 2630 Sugitani, Toyama, 930-0194 Japan

**Keywords:** Biological techniques, Cancer, Cell biology, Drug discovery

## Abstract

Monoclonal antibodies targeting the epidermal growth factor receptor (EGFR), including cetuximab and panitumumab, have been used in clinic settings to treat cancer. They have also recently been applied to antibody–drug conjugates (ADCs); however, their clinical efficacy is limited by several issues, including lower internalization efficiency. The binding of cetuximab to the extracellular domain of EGFR suppresses ligand-induced events; therefore, we focus on ligand-independent non-canonical EGFR endocytosis for the delivery of ADCs into cells. Tumor necrosis factor-α (TNF-α) strongly induces the endocytosis of the cetuximab-EGFR complex within 15 min via the p38 phosphorylation of EGFR in a tyrosine kinase-independent manner. A secondary antibody conjugated with saporin, a ribosome-inactivating protein, also undergoes internalization with the complex and enhances its anti-proliferative activity. Anti-cancer agents, including cisplatin and temozolomide, also induce the p38-mediated internalization. The results of the present study demonstrate that synchronous non-canonical EGFR endocytosis may be a feasible strategy for promoting the therapeutic efficacy of EGFR-targeting ADCs in clinical settings.

## Introduction

The aberrant activation of the epidermal growth factor receptor (EGFR), a member of the HER/ErbB family of receptor tyrosine kinases (RTKs), by its overexpression or mutations is a major mechanism underlying the development and progression of human malignant tumors, including colorectal cancer, lung cancer, and glioblastoma multiforme (GBM)^1–4^. Various EGFR-targeted agents, including tyrosine kinase inhibitors (TKIs) and neutralizing monoclonal antibodies (mAbs), have been developed for the treatment of EGFR-driven cancers^5,6^. Although the clinical efficacy of these agents has been demonstrated, the rapid establishment of acquired resistance via secondary *EGFR* mutations or the activation of proliferative/survival bypass pathways has also been reported^7–10^. Therefore, the development of new therapeutic strategies that overcome intrinsic and acquired resistance is desired.

Antibody–drug conjugates (ADCs), a new class of targeted agents, consisting of mAbs, cytotoxic payloads, and linkers that conjugate the two components, have potential as selective molecular-targeted agents for cancer therapy^11,12^. ErbB receptors as membrane-expressing target antigens have been the focus of research. Typical examples of successful development are the anti-HER2/ErbB2 ADCs, trastuzumab emtansine (T-DM1) and trastuzumab deruxtecan (T-DXd), for HER2-positive breast and gastric cancers^12–15^. Although vigorous attempts have been made to develop EGFR-targeting ADCs, those that release payloads intracellularly have not yet been successful^16,17^. The findings of these clinical trials suggest that the balance between the non-selective toxicity of the payload and the effects exerted in cancer cells are important. Therefore, increases in the internalization efficiency of EGFR-targeting ADCs may enhance their anti-tumor activities.

Extensive evidence has been obtained to show the intracellular trafficking of EGFR^18–21^. The ligand-bound activated EGFR dimer rapidly undergoes endocytosis via two major internalization routes, clathrin-mediated endocytosis (CME) and clathrin-independent endocytosis^22–26^. The endocytosed EGF-EGFR complex is transported to early endosomes, and is then sorted to either degradation or recycling pathways. We recently demonstrated that EGF induced the non-canonical CME of ligand-unoccupied and kinase-inactive EGFR monomers via the activation of downstream p38 in parallel with the canonical CME of ligand-bound EGFR active dimers^22,23^. A similar non-canonical mechanism is also driven by cellular stress-inducing agents, including tumor necrosis factor-α (TNF-α) and cisplatin, in which p38 phosphorylates EGFR at the Ser/Thr cluster around the clathrin-binding region^27–33^. However, these mechanisms have yet to be investigated for the internalization of EGFR-targeted ADCs.

Therefore, the present study investigated whether the non-canonical CME of EGFR promoted the internalization of membrane-bound EGFR-targeted mAbs. The results obtained demonstrated that the mAb-EGFR complex was markedly and synchronously internalized via the p38-mediated phosphorylation of EGFR upon a stimulation with TNF-α and cytotoxic anti-cancer agents. Based on these results, we examined the effects of a toxin-conjugated secondary antibody in cetuximab- and TNF-α-treated human cancer cells in vitro.

## Results

### Cetuximab inhibits EGF-induced EGFR endocytosis

**Figure 1:**
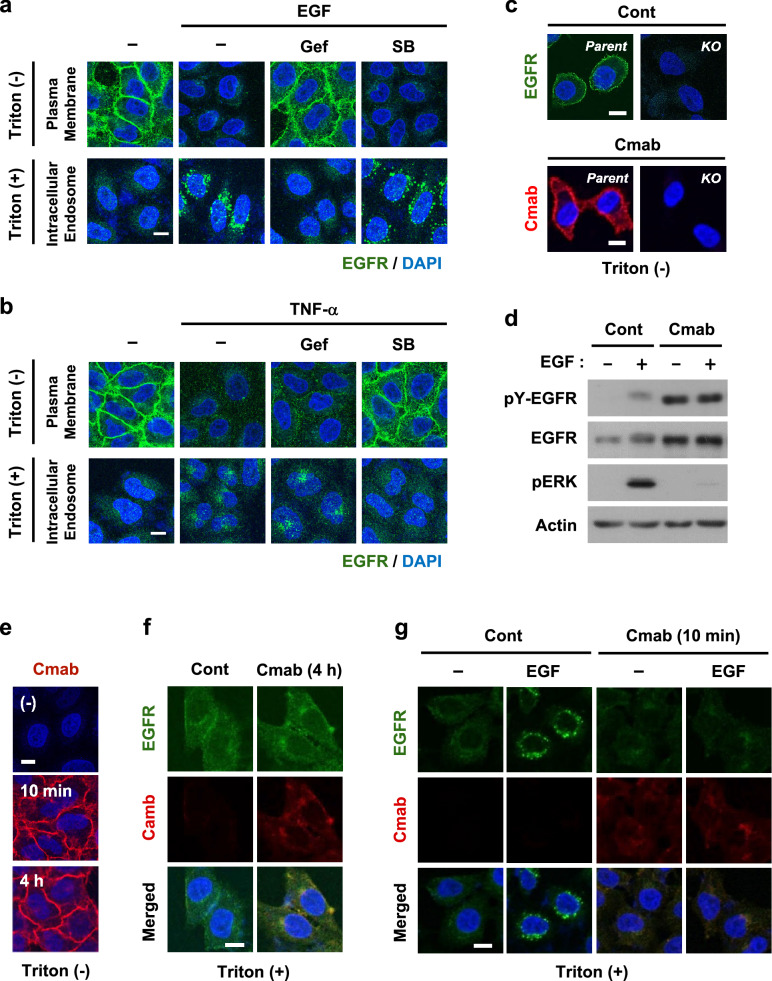
Cetuximab inhibits EGF-induced EGFR endocytosis. (**a**,**b**) HeLa cells were pretreated with 1 µM gefitinib or 10 µM SB203580 for 30 min, and then stimulated with 100 ng/mL EGF (**a**) or 20 ng/mL TNF-α (**b**) for another 15 min. The localization of EGFR on the cell surface (upper panel) or in the cytoplasm (lower panel) was investigated by immunofluorescence. DAPI, 4′,6-diamidino-2-phenylindole. (**c**) Parent HeLa cells and EGFR-knockout (KO) HeLa cells were pretreated with 100 nM cetuximab (Cmab) for 10 min. The cell surface expression of EGFR and Cmab was investigated by immunofluorescence. (**d**) Whole-cell lysates were prepared from HeLa cells stimulated with EGF for 10 min. The expression of the indicated proteins was detected by immunoblotting. pY-EGFR shows Tyr-1068-phosphorylated EGFR. Original blots are published in Supplementary Fig. S3. (**e**) HeLa cells were pretreated with 100 nM Cmab for 10 min or 4 h, and then its surface expression was investigated by immunofluorescence. (**f**,**g**) HeLa cells were treated with Cmab for 4 h (**f**), or pretreated with cetuximab for 10 min and then stimulated with EGF for another 15 min (**g**). The localization of EGFR and cetuximab in the cytoplasm was investigated by immunofluorescence. Scale bar = 10 µm.

In immunofluorescence staining, permeabilization with 0.5% Triton X-100 largely lost the membrane staining of EGFR, but its intracellular staining was unaffected compared to the standard concentration of 0.1% (Supplementary Fig. S1). Therefore, we used non-permeabilized (0%) and permeabilized (0.5%) conditions to separately detect the cell surface and intracellular EGFR, respectively (Fig. 1). A stimulation with both EGF and TNF-α for 15 min strongly induced the internalization of EGFR, which was confirmed by its decreased expression on the plasma membrane and corresponding redistribution to intracellular endosomes, indicating highly efficient endocytosis (Fig. 1a,b). Both internalization events appeared to be similar; however, the underlying mechanisms were different. Gefitinib, an EGFR-TKI, and SB203580, a p38 inhibitor, selectively blocked EGF- and TNF-α-induced endocytosis, respectively, indicating the existence of two independent internalization mechanisms: EGF-induced canonical endocytosis and TNF-α-induced non-canonical endocytosis.

We then focused on the internalization of cetuximab, a clinically applied anti-EGFR-neutralizing mAb. The specific binding of cetuximab to surface EGFR was confirmed using *EGFR*-KO HeLa cells (Fig. 1c). In addition, cetuximab inhibited EGF-induced ERK activation, but induced the autophosphorylation of EGFR at Tyr-1068 (Fig. 1d, Supplementary Figs. S2a and S3). The incubation of HeLa, A549 and DLD1 cells with cetuximab for up to 24 h induced a slight internalization of EGFR and cetuximab, indicating that the spontaneous internalization was less efficient than we expected. (Fig. 1e,f and Supplementary Fig. S2b,c). Furthermore, a stimulation with EGF did not induce the internalization of cetuximab, it instead inhibited ligand-induced EGFR endocytosis (Fig. 1g), indicating that the canonical system is not available for the intracellular delivery of anti-EGFR mAbs.

### Cellular stress-induced internalization of anti-EGFR antibodies

**Figure 2:**
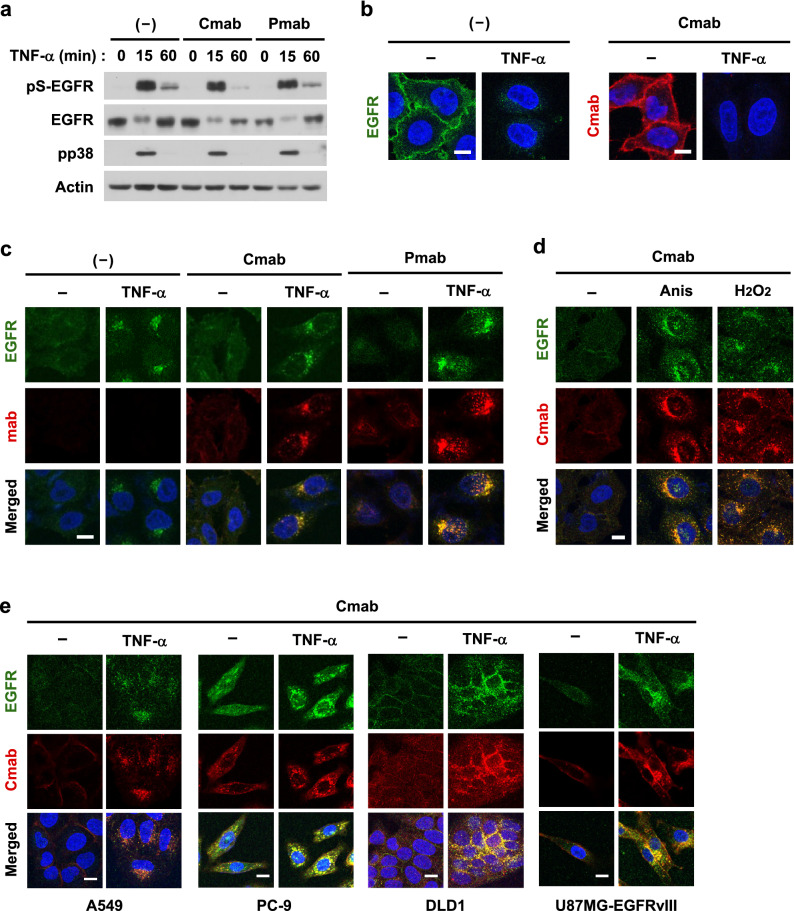
Internalization of anti-EGFR antibodies by cellular stress. (**a**) HeLa cells were pretreated with 100 nM cetuximab (Cmab) or 30 nM panitumumab (Pmab) for 10 min and then stimulated with 20 ng/mL TNF-α for 15 or 60 min. The expression of the indicated proteins was detected by immunoblotting. pS-EGFR shows Ser-1015-phosphorylated EGFR. Original blots are published in Supplementary Fig. S3. (**b**,**c**) HeLa cells were pretreated with cetuximab or panitumumab for 10 min and then stimulated with TNF-α for 15 min. (**d**) HeLa cells were pretreated with cetuximab for 10 min and then stimulated with 50 µM anisomycin for 30 min or 300 µM H_2_O_2_ for 15 min. (**e**) A549, PC-9, DLD1, and U87MG-EGFRvIII cells were pretreated with cetuximab for 10 min and then stimulated with TNF-α for 15 min. The expression of EGFR and cetuximab/panitumumab on the cell surface (**b**) and in intracellular compartments (**c**–**e**) was investigated by immunofluorescence. Scale bar = 10 µm.

We then attempted to apply the non-canonical EGFR endocytosis system to the internalization of membrane-bound anti-EGFR antibodies. We previously demonstrated that the phosphorylation of a Ser/Thr cluster of EGFR around the binding site of the clathrin adaptor protein AP-2, consisting of a di-leucine motif (Leu-1010/1011), induced the non-canonical CME of EGFR^22,34–36^. Pretreatment with cetuximab for 30 min was enough to its selective binding to the cell surface. Therefore, we investigated the effects of cetuximab on the TNF-α-induced phosphorylation of EGFR at Ser-1015, a typical site inducing CME. The binding of cetuximab or panitumumab to the extracellular domain of EGFR did not affect TNF-α-induced Ser-1015 phosphorylation (pS-EGFR), which resulted in the successful endocytosis of the EGFR-mAb complex (Fig. 2a and Supplementary Fig. S4). Cell surface EGFR and cetuximab both almost completely disappeared upon the stimulation with TNF-α for 15 min (Fig. 2b). In contrast, they appeared in intracellular endosomes in TNF-α-treated cells, and their localization completely overlapped (Fig. 2c and Supplementary Fig. S2c). The effectiveness of TNF-α in inducing endocytosis was similar to that of cells that were pretreated with cetuximab for 4 h (Supplementary Fig. S2e). Panitumumab was also efficiently internalized by the stimulation with TNF-α (Fig. 2c). Other cellular stressors, including anisomycin and hydrogen peroxide, induced the internalization of EGFR and cetuximab (Fig. 2d). Furthermore, TNF-α-induced internalization was commonly observed in various human cancer cells, including non-small cell lung cancer (A549 and PC-9), colorectal cancer (DLD1) and glioblastoma (U87MG expressing EGFRvIII mutant), while spontaneous internalization was occurred efficiently in PC-9 cells (Fig. 2e). The disappearance of cetuximab from the cell surface by TNF-α stimulation was cell-type dependent, with a small amount remaining on the cell surface in A549 cells and most in DLD1 cells (Supplementary Fig. S2d). Collectively, cellular stress conditions induced the efficient and synchronous delivery of the surface EGFR-mAb complex to the cytoplasmic region around the nucleus.

### Role of p38 in the internalization of cetuximab

**Figure 3:**
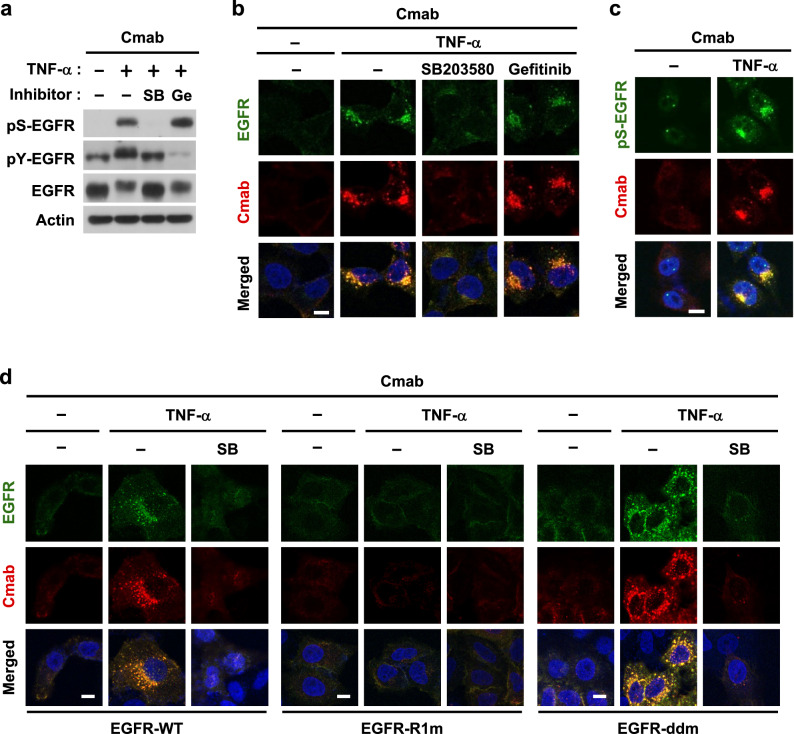
The p38-dependent intracellular trafficking of anti-EGFR antibodies. (**a**,**b**) HeLa cells were pretreated with 1 µM gefitinib or 10 µM SB203580 for 30 min. After a further pretreatment with 100 nM cetuximab for 10 min, cells were stimulated with 20 ng/mL TNF-α for 15 min. The expression of the indicated proteins (**a**) and the cell surface expression of both EGFR and cetuximab (**b**) were analyzed by immunoblotting and immunofluorescence, respectively. Original blots are published in Supplementary Fig. S3. (**c**) HeLa cells were pretreated with cetuximab for 10 min and then stimulated with TNF-α for 15 min. (**d**) EGFR-knockout HeLa cells re-expressing wild-type and mutant EGFR (WT, R1m and ddm) were pretreated with SB203580 for 30 min, cetuximab for 10 min, and then stimulated with TNF-α for 15 min. The localization of pS-EGFR, EGFR, and cetuximab in the cytoplasm was investigated by immunofluorescence. Scale bar = 10 µm.

The non-canonical endocytosis of EGFR was previously shown to be regulated by p38^27^; therefore, we attempted to clarify the role of p38 in the internalization of cetuximab. In the presence of cetuximab, SB203580, but not gefitinib, inhibited TNF-α-induced pS-EGFR expression (Fig. 3a and Supplementary Fig. S4) and the subsequent internalization of the cetuximab-EGFR complex (Fig. 3b). In addition, immunofluorescence staining revealed that pS-EGFR co-localized with cetuximab, indicating that endocytosed EGFR in the complex was phosphorylated by p38 (Fig. 3c). We also demonstrated the involvement of a non-canonical mechanism using *EGFR*-KO HeLa cells reconstituted with wild-type EGFR or mutants (Supplementary Fig. S3). The mutant R1m has amino acid substitutions at non-canonical phosphorylation sites; therefore, it inhibits non-canonical mechanisms. In contrast, the dimer-deficient mutant (ddm) has deletions and substitutions in the extracellular and intracellular regions required for ligand binding and dimerization and, thus, only drives the non-canonical mechanism. Figure 3d shows the internalization of the cetuximab-EGFR complex in the wild type and ddm mutant, but not in the R1m mutant. Therefore, the cetuximab-EGFR complex underwent p38-mediated phosphorylation, followed by non-canonical endocytosis.

### Trafficking route of the cetuximab-EGFR complex

**Figure 4:**
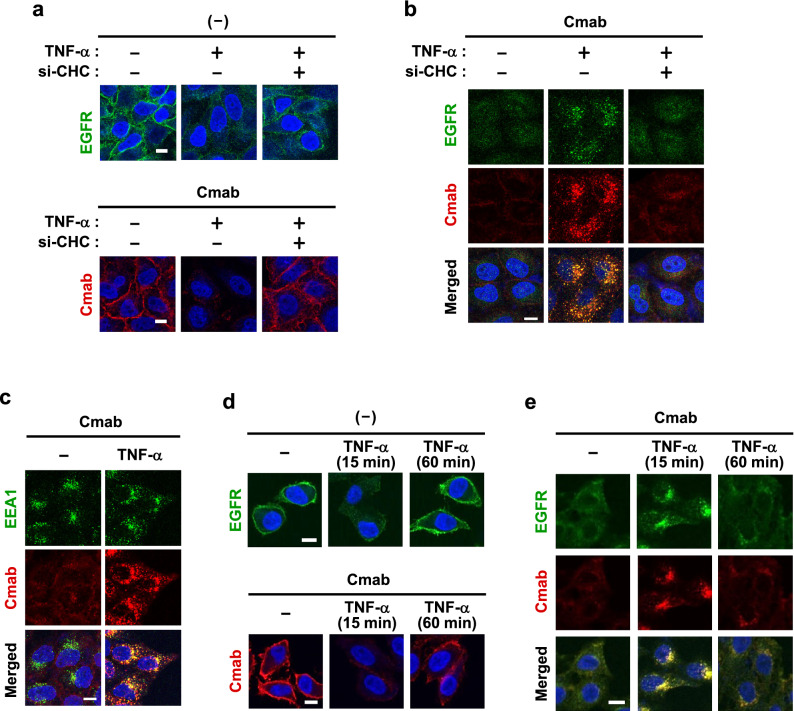
The trafficking route of the cetuximab-EGFR complex. (**a**,**b**) HeLa cells were transfected with siRNA for the negative control (−) or the clathrin heavy chain (si-CHC). After a 72-h incubation, cells were pretreated with 100 nM cetuximab for 10 min and then stimulated with 20 ng/mL TNF-α for 15 min. The cell surface (**a**) and intracellular localization (**b**) of EGFR and cetuximab was investigated by immunofluorescence. (**c**) HeLa cells were pretreated with 100 nM cetuximab for 10 min and then stimulated with 20 ng/mL TNF-α for 15 min. Early endosome antigen 1 (EEA1) and cetuximab in the cytoplasm were stained. (**d**,**e**) HeLa cells were pretreated with 100 nM cetuximab for 10 min and then stimulated with 20 ng/mL TNF-α for 15 or 60 min. The localization of EGFR and cetuximab on the cell surface (**d**) or in the cytoplasm (**e**) was investigated by immunofluorescence. Scale bar = 10 µm.

After internalization via CME, EGFR is sorted to early endosomes, and is finally recycled back to the plasma membrane without lysosomal degradation^22,29,32,33^. Therefore, we herein investigated the endocytic trafficking of the cetuximab-EGFR complex after internalization. We initially demonstrated that the complex was internalized via CME because the RNAi knockdown of CHC completely inhibited TNF-α-induced internalization (Fig. 4a,b). In addition, endocytosed cetuximab co-localized with EEA1, an early endosome marker (Fig. 4c), after 15 min of the stimulation, and was then recycled back to the plasma membrane together with EGFR 60 min after the stimulation (Fig. 4d,e). These results demonstrated that cetuximab was sorted to early endosomes and recycling endosomes in the complex with EGFR and was eventually recycled to the plasma membrane.

### Significant increase in ADC activity in vitro

**Figure 5:**
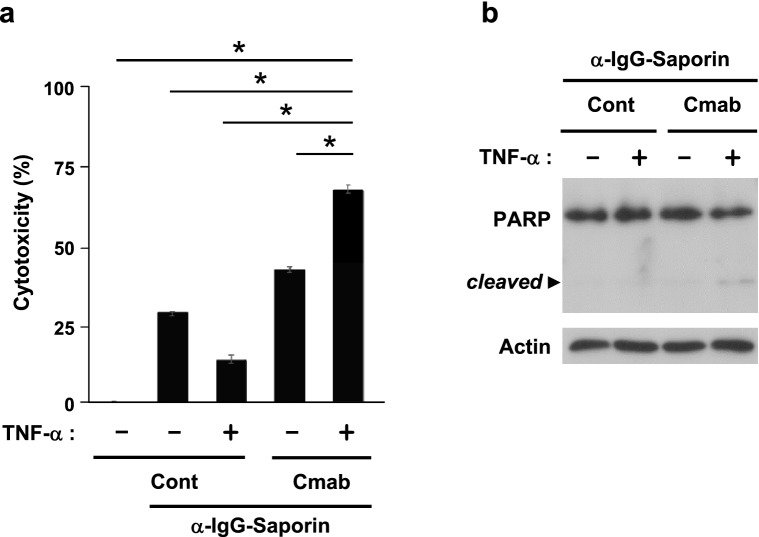
Effects of a second immunotoxin on cell proliferation. (**a**,**b**) HeLa cells were pretreated with 100 nM cetuximab and 5 nM saporin-conjugated anti-human IgG antibody (α-IgG-Saporin) for 30 min and then stimulated with 20 ng/mL TNF-α for 24 h. The cytotoxicity (**a**) and cleavage of PARP (**b**) were analyzed by the WST-8 assay and immunoblotting, respectively. Cytotoxicity was calculated using the values of cell proliferation in the absence of the trimer complex. Original blots are published in Supplementary Fig. S3. Values are shown as the mean ± SD. *p < 0.01.

To investigate the effects of antibody internalization on cell proliferation, we used the second immunotoxin. The formation and endocytosis of the trimer complex of EGFR, cetuximab, and the immunotoxin delivered saporin into cells. HeLa cells were pretreated with the complex for 30 min and then incubated with TNF-α for 24 h. As shown in Fig. 5a, cytotoxicity was significantly increased by the trimer complex, and cleaved PARP was detected in an immunoblot analysis (Fig. 5b and Supplementary Fig. S4). These results demonstrated that the non-canonical endocytosis of EGFR enhanced the ADC activity of anti-EGFR antibodies.

### Anti-cancer agents induce the internalization of cetuximab

**Figure 6:**
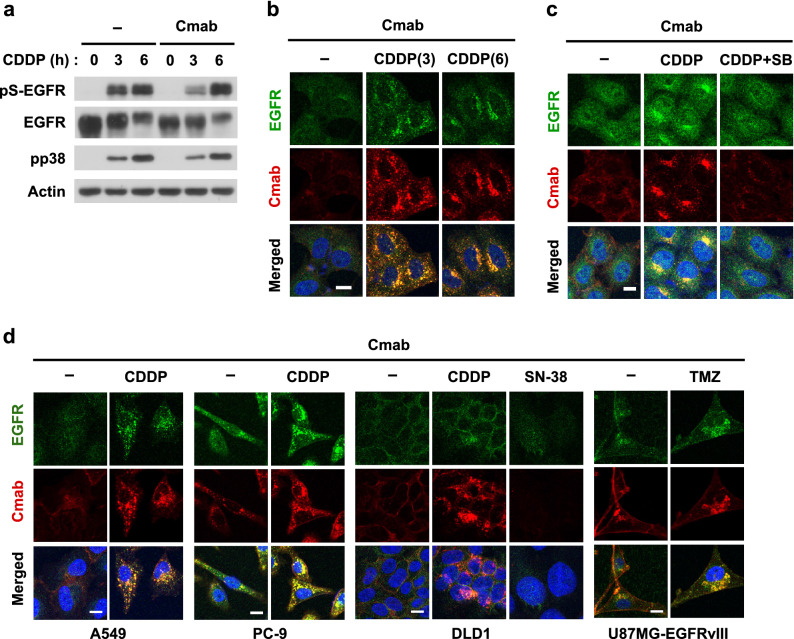
Sustained internalization of anti-EGFR antibodies by cisplatin. (**a**,**b**) HeLa cells were pretreated with 100 nM cetuximab for 10 min and then stimulated with 100 µM cisplatin (CDDP) for 3 or 6 h. The expression of phospho-p38 and pS-EGFR was examined by immunoblotting (**a**). The intracellular expression of EGFR and cetuximab was investigated by immunofluorescence (**b**). Original blots are published in Supplementary Fig. S3. (**c**) HeLa cells were pretreated with 10 µM SB203580 for 30 min, treated with 100 nM cetuximab for 10 min, and then stimulated with 100 µM CDDP for 6 h. The intracellular expression of EGFR and cetuximab was investigated by immunofluorescence. (**d**) A549, PC-9, DLD1, and U87MG-EGFRvIII cells were pretreated with 100 nM cetuximab for 10 min and then stimulated with 100 µM CDDP for 6 h, 1 µM SN-38 for 48 h, or 100 µM temozolomide (TMZ) for 48 h. Intracellular EGFR and cetuximab were visualized by immunofluorescence. Scale bar = 10 µm.

We investigated the potential of p38 activators other than TNF-α for use in clinical settings. We focused on the clinically available agents, cisplatin and temozolomide because they are known to trigger the continuous non-canonical endocytosis of EGFR^27,31,37^. As shown in Fig. 6a and Supplementary Fig. S4, cisplatin induced the prolonged phosphorylation of EGFR at Ser-1015 for a minimum of 6 h in HeLa cells, and this correlated with the stable internalization of cetuximab in a p38-dependent manner (Fig. 6b,c). In addition, cisplatin, but not SN-38, was effective in lung and colorectal cancer cells (Fig. 6d). Furthermore, temozolomide, an alkylating agent that is clinically used to treat GBM, induced the endocytosis of the cetuximab-EGFRvIII mutant in U87MG cells (Fig. 6d). These results suggested that clinically applied chemotherapeutic agents are available as inducers of the endocytosis of ADCs in each tumor type.

## Discussion

The non-canonical regulation of ligand-unbound, tyrosine kinase-inactive EGFR is being increasingly recognized as a significant advance in EGFR biology^22,23,26,27,29,38^. The most characterized non-canonical intracellular trafficking pathway is the p38-mediated endocytic recycling pathway, which is clearly different from clathrin-independent fast endocytosis-mediated endocytosis (FEME)^39^. In the present study, endocytic sorting was applied to ADC therapy, and the results obtained demonstrated that TNF-α induced the synchronous intracellular delivery of the cetuximab/panitumumab-EGFR complex via p38-dependent CME. This protocol makes it possible to rapidly reach a peak payload concentration if the drug is quickly released from antibodies. In contrast, early recycling within 60 min limits the release of payloads. Therefore, the selection of appropriate linkers, such as pH-sensitive linkers that function in sorting endosomes with weak acidity, is the key to accomplish this purpose^40,41^. On the other hand, we propose another option for the prolonged intracellular accumulation of ADCs by chemotherapeutic agents, including cisplatin and temozolomide, which is beneficial because they are already used in clinical settings^27,28,37^. Moreover, irradiation, another p38-activating therapeutic, has been shown to induce the non-canonical endocytosis of EGFR^38,42^. Therefore, significant ADC activity may be achieved by establishing the optimum combination of a linker and p38 activator. Furthermore, a more detailed understanding of the recycling mechanism will lead to alternative strategies being proposed for efficient payload release from anti-EGFR antibodies. For example, the inhibition of sorting to recycling endosomes directs ADCs towards lysosomal degradation.

Similar to our strategy, it has been reported that intracellular delivery of cetuximab is enhanced by other inducers. Anti-human IgG, for example, strongly induces internalization of the cetuximab-EGFR complex by micropinocytosis, a clathrin-independent mechanism^43^. In addition, tumor uptake and therapeutic efficacy of EGFR-targeted ADCs are enhanced by cholesterol sequestration with nystatin^44^. It is interesting that nystatin induces p38 activation^45^; therefore, there is the possibility that endocytosis of EGFR-targeted antibody by cholesterol sequestration is mediated by the non-canonical mechanism. It is expected that increasing the efficiency of intracellular recruitment of ADC agents will lead to the development of new therapeutic strategies.

Photoimmunotherapy using cetuximab-sarotalocan (RM-1929), a new ADC harboring a light-activatable dye (IRDye 700DX; IR700), has recently been approved for the treatment of unresectable locally advanced or locally recurrent head and neck squamous cell carcinomas (HNSCCs) in Japan^46–48^. The activation of IR700 with red light damages the membrane integrity of cells, resulting in photo-sensitive cell death^49^. It also leads to the local and systemic induction of immunity. In this case, payload release is not required; however, the cytotoxicity of photo-activated IR700 may be strongly influenced by the site at which it is activated in the cell. Therefore, it is important to confirm whether the accumulation of the dye around the nucleus by non-canonical CME enhances its anti-cancer activity. Cisplatin is a standard chemotherapeutic agent for HNSCCs; therefore, further studies are needed to clarify the efficacy of cetuximab-sarotalocan by the pre-dosing of cisplatin just before photo-sensitization.

HER2-targeted ADCs, including T-DM1 and T-DXd, are used to treat metastatic HER2-positive breast and gastric cancers^13^. In addition, clinical trials on patritumab deruxtecan (U3-1402/HER3-DXd) are now underway for patients with breast, lung, and colorectal cancers^50,51^. However, since the Ser/Thr residues inducing the non-canonical endocytosis of EGFR are not conserved in HER2 or HER3, their internalization rates are poor. Therefore, the critical mechanisms underlying endocytosis for ADC therapy need to be elucidated in more detail. Melatonin, a hormone secreted by the pineal gland, was recently show to significantly induce the endocytosis of HER2 and potentiate the cytotoxic effects of neratinib, a next-generation irreversible TKI of EGFR and HER2^52^. In addition, osimertinib, a third-generation EGFR-TKI, enhanced the cellular uptake and antitumor activity of HER3-DXd in EGFR-mutant non-small cell lung cancers^53^. The integration of these findings and information showing that EGFR forms heterodimers with HER2 and HER3 will contribute to the future establishment of an efficient ADC strategy that targets EGFR/HER family receptors.

In summary, based on our recent contributions to EGFR biology, we provide a novel strategy for the efficient intracellular delivery of anti-EGFR antibodies. Although several EGFR-targeted ADCs have been developed, those that exert their effects by releasing the payload from antibodies have not yet been approved. Even though many studies have been conducted on the selection of antibodies, linkers, and anticancer compounds, further research is warranted to increase the intracellular payload concentration per ADC dose, which will attenuate the adverse effects/anti-cancer activity balance and lead to the successful development of EGFR-targeted ADCs.

## Methods

### Antibodies and reagents

Antibodies against phospho-p38 (Thr-180/Tyr-182), phospho-ERK (Thr-202/Tyr-204), phospho-EGFR (Tyr-1068), early endosome antigen 1 (EEA1) (C45B10), and clathrin heavy chain (CHC) (D3C6) were purchased from Cell Signaling Technology (Danvers, MA). Anti-EGFR (1005) and anti-actin (C-11) antibodies were obtained from Santa Cruz Biotechnology (Santa Cruz, CA, USA). A phospho-EGFR (Ser-1015) rabbit recombinant antibody was generated as previously reported^22^. The clones LA1 (Merck KGaA, Darmstadt, Germany) and A-10 (Santa Cruz Biotechnology) were used in the immunofluorescence staining of EGFR on the cell surface and in the cytoplasm, respectively. Cetuximab and panitumumab were obtained from MedChemExpress (Monmouth Junction, NJ, USA) and BioVision (Milpitas, CA, USA), respectively. Recombinant human EGF and TNF-α were obtained from R&D Systems (Minneapolis, MN, USA). Anisomycin, cisplatin, gefitinib, hydrogen peroxide, and temozolomide were purchased from FUJIFILM Wako Pure Chemical Corporation (Osaka, Japan). SB203580 and SN-38 were obtained from Merck KGaA and BLD Pharmatech (Cincinnati, OH, USA), respectively. Hum-ZAP, a second immunotoxin that uses an affinity-purified goat anti-human IgG conjugated with the ribosome-inactivating protein saporin, was obtained from Advanced Targeting Systems (Carlsbad, CA, USA). All chemical inhibitors were dissolved in dimethyl sulfoxide (DMSO), and the final concentration of DMSO was less than 0.1%.

### Cell culture

HeLa cells were obtained from the American Type Culture Collection (ATCC, Rockville, TX, USA) and maintained in Dulbecco’s modified Eagle’s medium (DMEM) (high-glucose; Nissui Pharmaceutical, Tokyo, Japan) supplemented with 10% fetal calf serum, 4 mM l-glutamine, 100 U/mL penicillin, and 100 U/mL streptomycin (Meiji Seika Pharma, Tokyo, Japan) at 37 °C in 5% CO_2_. Human U87MG glioblastoma cells that overexpress EGFRvIII were provided by Motoo Nagane (Kyorin University, Japan) and maintained in DMEM supplemented with 10% fetal calf serum, 2 mM L-glutamine, 100 U/mL penicillin, and 100 μg/mL streptomycin. A549 (ATCC), DLD-1 (ATCC), and PC-9 cells (a kind gift from Prof. Kiura, Okayama University, Japan) were maintained in RPMI-1640 (Nissui Pharmaceutical) supplemented with 10% fetal calf serum, 2 mM glutamine, 100 U/mL penicillin, and 100 µg/mL streptomycin at 37 °C in 5% CO_2_.

### Immunoblotting

Whole cell lysates were prepared as previously described^54^, resolved by SDS-PAGE, and transferred to an Immobilon-P nylon membrane (Merk KGaA). The membrane was treated with Block Ace (KAC, Hyogo, Japan), then cut to the appropriate size and incubated with the primary antibodies described above. Antibodies were detected using horseradish peroxidase-conjugated anti-rabbit or mouse immunoglobulin G (Dako, Agilent Technologies, Santa Clara, CA, USA) and visualized with an enhanced chemiluminescence system (Thermo Fisher Scientific). Some antibody reactions were performed in Can Get Signal solution (Toyobo, Tokyo, Japan). Uncropped scans of the original blots were supplied in Supplementary Fig. S3.

### RNA interference

A small interfering RNA (siRNA) against CTLC encoding CHC (HSS102017) and Stealth RNAi™ siRNA Negative Control Lo GC were purchased from Thermo Fisher Scientific (Waltham, MA, USA). HeLa cells were transfected with siRNAs at a final concentration of 50 nM using Lipofectamine 3000 (Thermo Fisher Scientific) according to the manufacture's instructions. Cells were used in experiments 72 h post-transfection.

### Immunofluorescence

Cells were seeded on coverslip glass (Matsunami Glass, Osaka, Japan). Two days after seeding, cells were incubated with inhibitors and ligands or transfected with siRNAs. Cells were rinsed in cold PBS and fixed in 4% paraformaldehyde at room temperature for 15 min. After fixation, cells were permeabilized in PBS containing 0.5% Triton X-100 and washed by PBS for intracellular EGFR staining. This step was omitted for cell surface staining. Cells were then incubated for 40 min with primary antibodies and incubated with isotype-specific secondary antibodies conjugated with Alexa Fluor (Thermo Fisher Scientific) for 30 min. These antibodies were diluted in PBS containing 0.5% BSA. Microscopy was performed using a Zeiss LSM 700 confocal microscope (Oberkochen, Germany).

### Establishment of mutant EGFR-expressing cells

EGFR-knockout (EGFR-KO) HeLa cells were generated by the genome-editing clustered regularly interspaced short palindromic repeat (CRISPR)/CRISPR-associated protein 9 (Cas9) system. A double-stranded DNA oligonucleotide encoding EGFR gRNA (5’-ATAACTGTGAGGTGGTCCTT-3’) was ligated into pSpCas9 (BB)-2A-Puro (PX495) v.2.0 (a gift from Dr. Feng Zhang; plasmid 62988; Addgene, Cambridge, MA, USA) using the method described by Ran et al.^55^. Cells were transfected with the plasmid DNA generated using Lipofectamine 3000 according to the manufacture's instructions. After 48 h of transfection, cells were cultured with 2 µg/mL puromycin (FUJIFILM Wako Pure Chemical Corporation) for 48 h, and single-cell clones were collected using the FACSAria SORP cell sorter (BD, Piscataway, NJ, USA). Mature clones were subjected to DNA sequencing and immunoblotting to confirm the deletion of EGFR expression (Supplementary Fig. S3a,b). EGFR-KO cells were rescued with pEGFP-N1 plasmid DNAs encoding *EGFR* (wild type, R1m; S1015A/T1017A/S1018A, and ddm; ΔCR1/I682Q/V924R) (Supplementary Fig. S3c,d). EGFR re-expressing cells were selected by 1 mg/mL G418, while clones were selected using the cell sorter.

### Cell viability assay

HeLa cells were seeded on a 96-well plate (5000 cells/well). After an overnight culture, cells were treated with cetuximab, the second immunotoxin, and TNF-α for 24 h. Cells were then subjected to the WST-8 cell counting assay (DOJINDO, Kumamoto, Japan) according to the manufacture's instructions for 2 h. Absorbance at 595 nm was measured using a microplate reader. Values are shown as the mean ± S.D. The significance of differences was assessed by the Tukey–Kramer test and *p < 0.01 was considered to be significant.

## Supplementary Information

Supplementary Figures.

## Data Availability

The datasets used and/or analysed during the current study available from the corresponding author on reasonable request.
