# Supplementary material for: Synchronous intracellular delivery of EGFR-targeted antibody–drug conjugates by p38-mediated non-canonical endocytosis
Source: Sci Rep. 2022 Jul 7;12:11561. doi: 10.1038/s41598-022-15838-8 (PMC9262980; doi:10.1038/s41598-022-15838-8)
Supplement: Supplementary file 1 — Supplementary Figures. [file 41598_2022_15838_MOESM1_ESM.pdf]

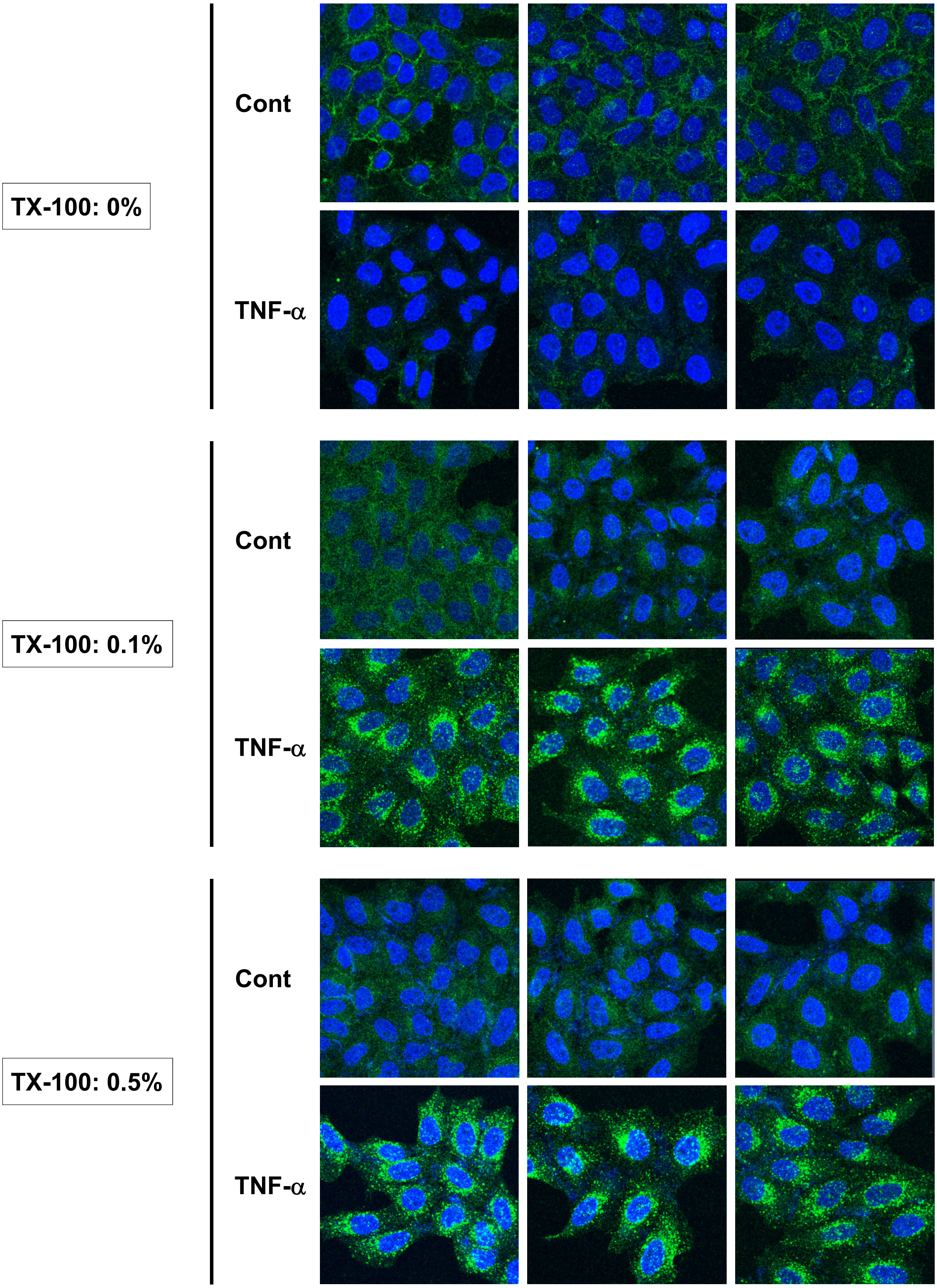

Supplementary Figure S1. HeLa cells were stimulated with TNF-α for 15 min. After permeabilization with 0, 0.1 or 0.5% Triton X-100, the localization of EGFR was investigated by immunofluorescence. Three independent fields were shown in each conditions.

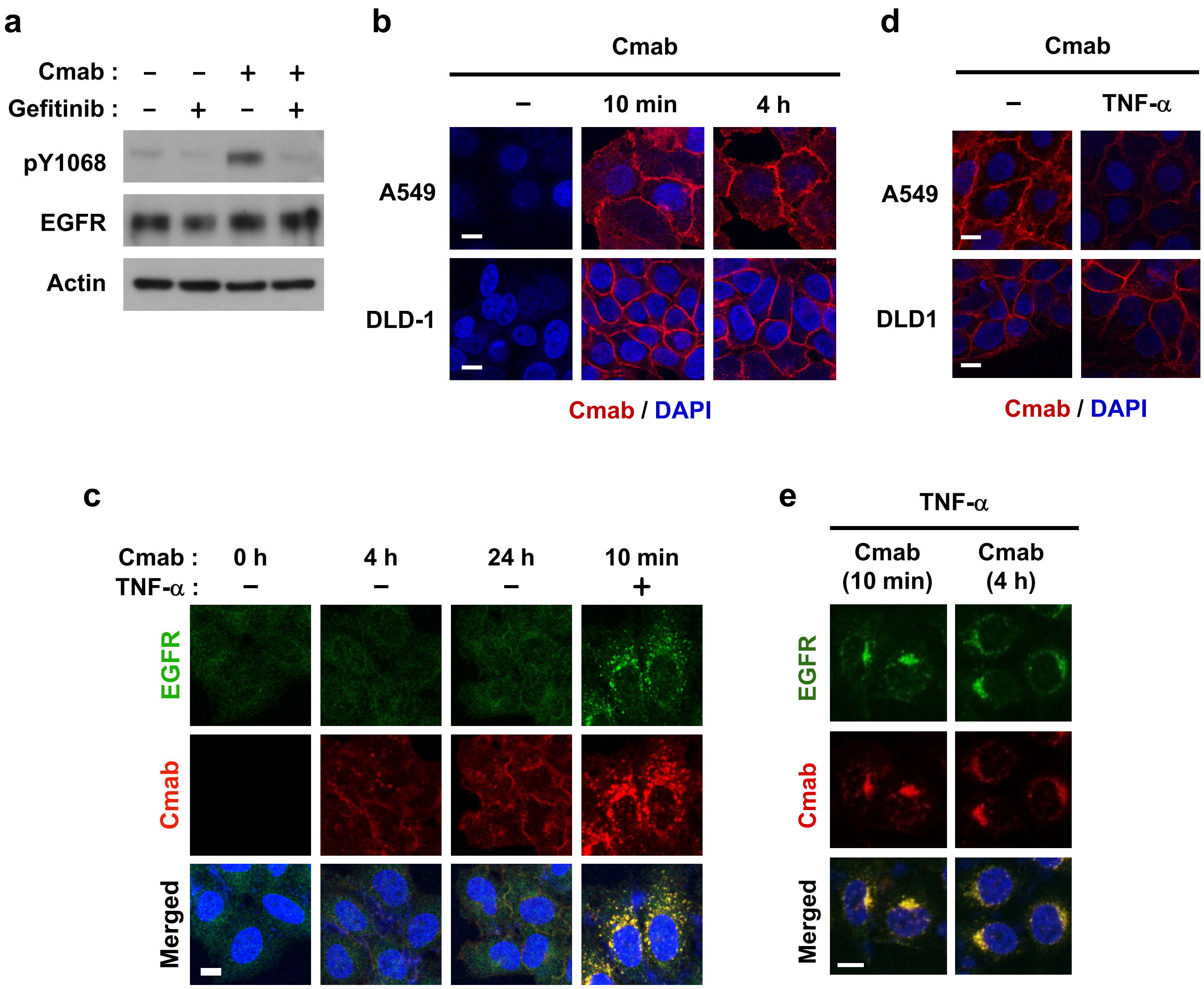

Supplementary Figure S2. **a,** HeLa cells were pretreated with 1 μM gefitinib for 30 min, and then stimulated with 10 nM cetuximab (Cmab) for another 15 min. The expression of Tyr-1068-phosphorylated EGFR (pY1068), total EGFR and Actin was detected by immunoblotting. Original blots are published in Supplementary Figure. S3. **b**, A549 and DLD1 cells were pretreated with 100 nM Cmab for 10 min or 4 h. **c**, HeLa cells were treated with 100 nM Cmab for 0, 4, 24 h or 10 min, and were stimulated with or without TNF-α for 15 min. **d**, A549 and DLD1 cells were pretreated with 100 nM Cmab for 10 min, and then stimulated with TNF-α for 15 min. **e,** HeLa cells were pretreated with 100 nM cetuximab for 10 min or 4 h, and then stimulated with TNF-α for 15 min. The localization of EGFR and Cmab on the cell surface (**b, d**) or in the cytoplasm (**c, e**) was investigated by immunofluorescence. Scale bar = 10 μm.

a

<u>Parent HeLa cells</u>

<Exons 1-3>

MRPSGTAGAALLALLAALCPASRALEEKKVCQGTSNKLTQLGTFEDHFLSLQRMFNNCEV
VLGNLEITYVQRNYDLSFLKTIQEVAGYVLIALNTVERIPLENLQIIRGNMYYENSYALA
VLSNYDANKTGLKELPMRNLQEILHGAVRFSNNPALCNVESIQWRDIVSSDFLSNMSMDF
QNHLGS

<u>EGFR-KO HeLa cells</u>

<1st allele>

MRPSGTAGAALLALLAALCPASRALEEKKVCQGTSNKLTQLGTFEDHFLSLQRMFNNCEV
VWEFGNYLCAEEL

<2nd allele>

MRPSGTAGAALLALLAALCPASRALEEKKVCQGTSNKLTQLGTFEDHFLSLQRMFNNCEV
AWGIWKLPMCRGIMIFPS

b

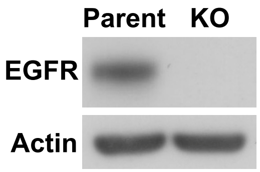

c

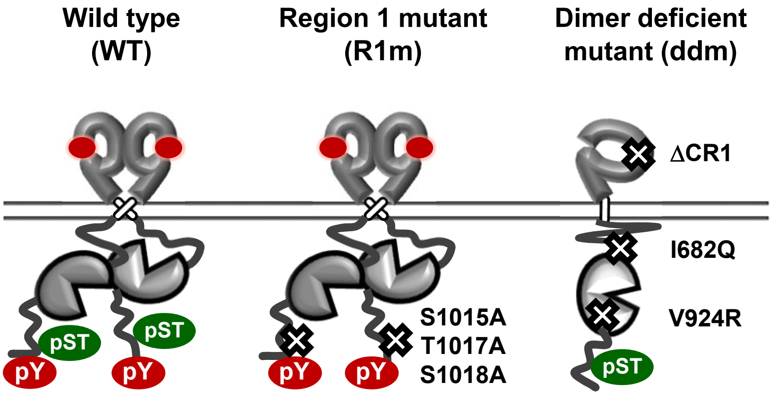

d

EGFR reactions	WT	R1m	ddm
EGF-induced activation			
EGF-binding	○	○	×
Dimerization	○	○	×
EGF-induced endocytosis			
Canonical	○	○	×
Noncanonical	○	×	×
TNF-α-induced endocytosis			
Noncanonical	○	×	○

Supplementary Figure S3. Establishment of *EGFR*-knockout (KO) HeLa cells by the CRISPR/Cas9 genome-editing system. **a,** Amino acid sequences encoded by human *EGFR* exons 1-3 in parent and *EGFR*-KO HeLa cells. Mutated amino acids in both alleles are shown in red. **b,** The expression of EGFR and Actin in parent and *EGFR*-KO cells was detected by immunoblotting. Original blots are published in Supplementary Figure. **c,** Schematic diagrams of wild type (WT), region 1 mutant (R1m) and dimer deficient mutant (ddm) of EGFR are shown. The mutant R1m has amino acid substitutions at non-canonical phosphorylation sites (S1015A, T1017A and S1018A). The mutant ddm has a deletion (ΔCR1) and point mutations (I682Q and V924R). **d,** Functions in each EGFR are summarized in the table.

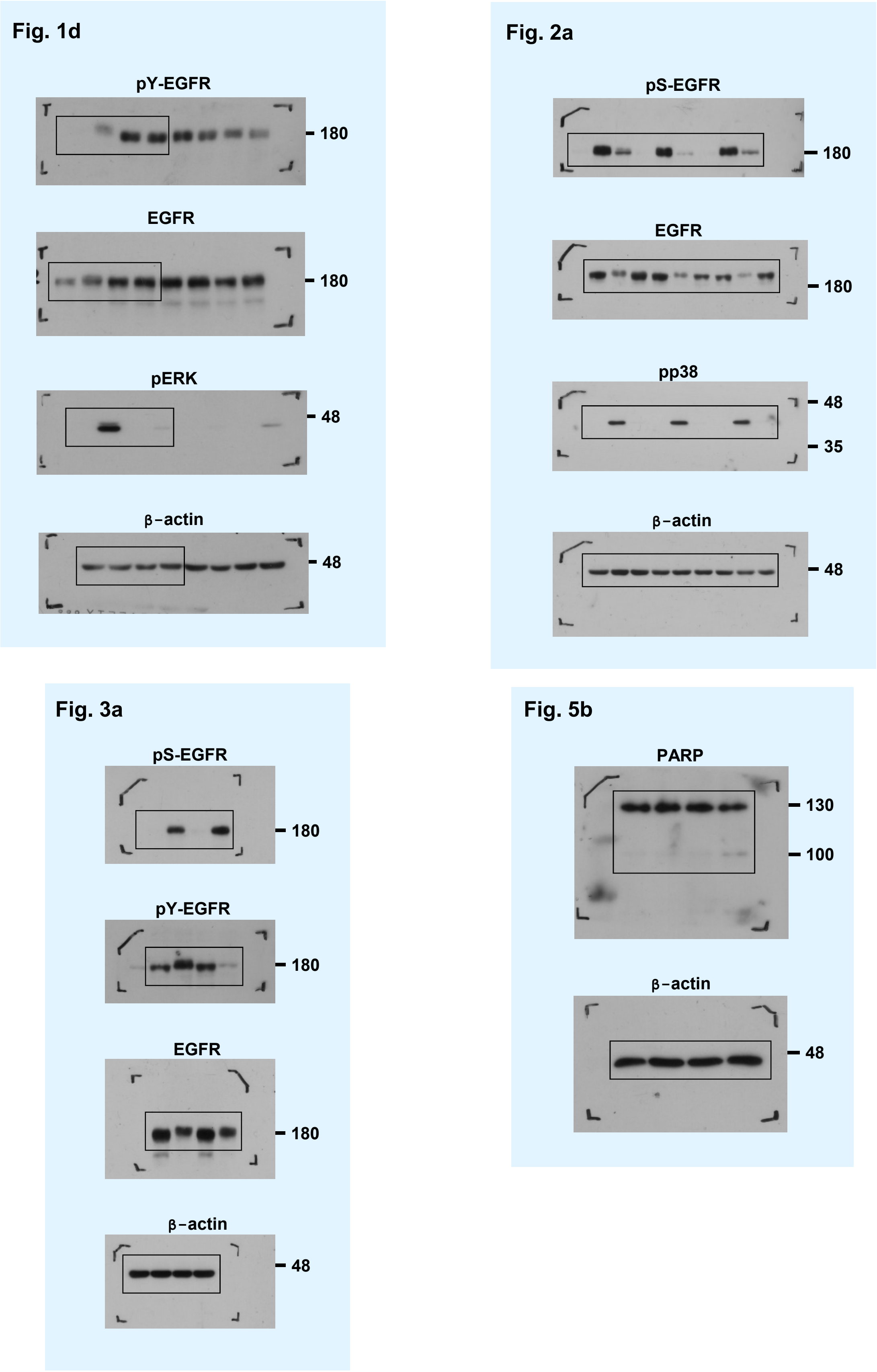

Supplementary Figure S4. Full scans of Immunoblot data. Membranes were cut to the appropriate size around the molecular weight of the interested proteins after blocking, and then incubated with the primary antibodies.

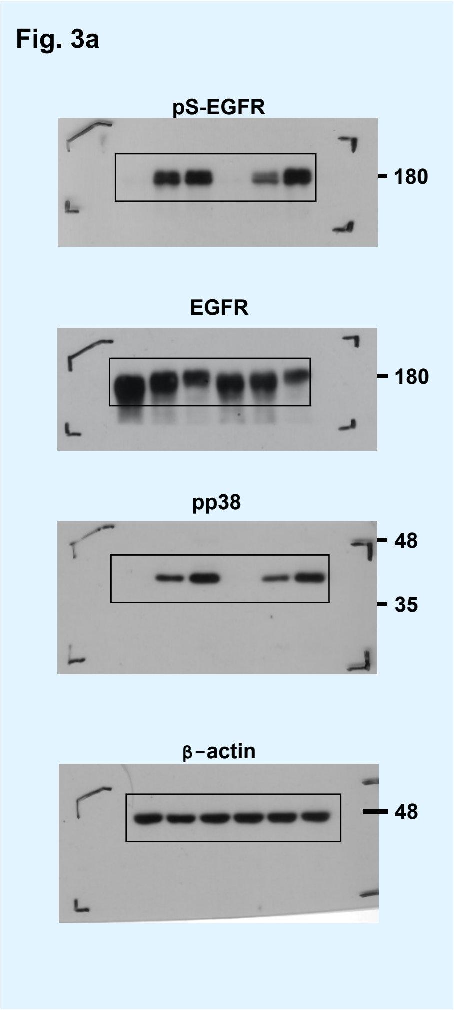

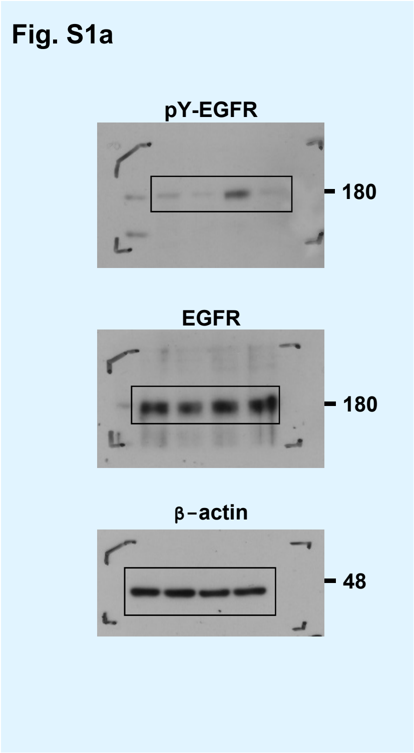

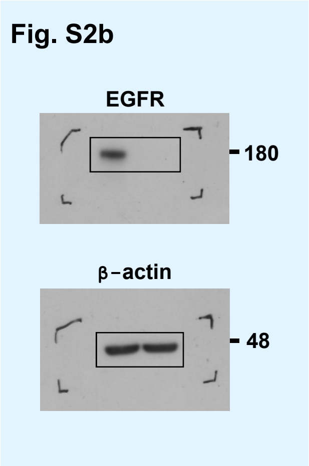

Supplementary Figure S4. Contiuned.
